# Asymptomatic SARS-CoV-2 Infection in Household Contacts of a Healthcare Provider, Wuhan, China

**DOI:** 10.3201/eid2608.201016

**Published:** 2020-08

**Authors:** Yi Luo, Edwin Trevathan, Zhengmin Qian, Yirong Li, Jin Li, Wei Xiao, Ning Tu, Zhikun Zeng, Pingzheng Mo, Yong Xiong, Guangming Ye

**Affiliations:** Zhongnan Hospital, Wuhan University, Wuhan, China (Y. Luo, Y. Li, J. Li, Z. Zeng, P. Mo, Y. Xiong, G. Ye);; Vanderbilt Institute for Global Health, Vanderbilt University Medical Center, Nashville, Tennessee, USA (E. Trevathan);; Saint Louis University, St. Louis, Missouri, USA (Z. Qian);; Central Hospital of Wuhan, Tongji Medical College, Huazhong University of Science and Technology, Wuhan (W. Xiao);; Wuhan University Remin Hospital, Wuhan (N. Tu).

**Keywords:** COVID-19, 2019 novel coronavirus disease, coronavirus disease, SARS-CoV-2, severe acute respiratory syndrome coronavirus 2, viruses, respiratory infections, zoonoses, asymptomatic, Wuhan, China, household contacts, healthcare workers

## Abstract

We found that all 5 asymptomatic household contacts of a Wuhan, China, physician with coronavirus disease had severe acute respiratory syndrome coronavirus 2 detected by PCR. The index patient and 2 contacts also had abnormal chest computed tomography scans. Asymptomatic infected household contacts of healthcare workers with coronavirus disease might be underrecognized.

Severe acute respiratory syndrome coronavirus 2 (SARS-CoV-2), the cause of the coronavirus disease (COVID-19) pandemic, is highly contagious and can put families of healthcare professionals at risk for both symptomatic COVID-19 and asymptomatic SARS-CoV-2 infection with potential to infect others (*1*–*4*). Data regarding asymptomatic SARS-CoV-2 infection (*5*) among families of healthcare professionals can help inform healthcare management and the public health response during the COVID-19 pandemic. We describe the case of a physician in Wuhan, China, who had mildly symptomatic COVID-19 and the subsequent asymptomatic SARS-CoV-2 infection in all 5 of his household contacts.

The index patient (patient 1) was a 39-year-old nephrologist at Central Hospital of Wuhan who had onset of a dry cough on January 31, 2020, was admitted with fever on February 7, and was diagnosed with symptomatic SARS-CoV-2 infection on February 10. During January 31–February 6, patient 1 lived with 5 other immediate family members, all of whom were hospitalized on February 11 at Zhongnan Hospital of Wuhan University for ethics committee–approved (approval no. 2019125) medical studies, for which informed consent was obtained. The household contacts were his 37-year-old wife, a laboratory physician without patient contact at Zhongnan Hospital (contact 1); 7-year-old fraternal twins, who were in contact only with family because of school closure and social distancing (contacts 2 and 3); a retired 62-year-old grandfather, who was a current smoker in good health (contact 4); and a retired 64-year-old grandmother in good health (contact 5).

All household contacts underwent chest computed tomography scans and throat swabs for quantitative real-time reverse transcription PCR (qRT-PCR) tests for SARS-CoV-2 nucleic acid, in addition to other routine laboratory examinations (Table). qRT-PCR tests on stool specimens of contacts 1, 2, and 3 were positive for SARS-CoV-2. Contact 1 also was positive for SARS-CoV-2 on qRT-PCR tests of multiple serial throat swab specimens but negative for SARS-CoV-2 on IgM and IgG tests. 

**Table:** Summary of laboratory results of a SARS-CoV-2–positive patient and 5 asymptomatic household contacts, Wuhan, China*

Laboratory test	Reference range	Patient 1	Contact 1	Contact 2†	Contact 3‡	Contact 4	Contact 5
C-reactive protein, mg/L	0–10	18.8	2.0	0.4	0.4	1.5	2.7
Leukocyte count, × 10^9^ cells/L	3.5–9.5	6.68	6.89	4.79	6.86	3.54	5.84
Lymphocyte ratio, %	20–50	17.70	18.50	45.50	67.90	34.60	33.10
CD19^+^ absolute count/μL	240–1317	140	147	626	767	271	299
ALT, U/L	7–45	45	11	520	16	15	7
AST, U/L	13–35	21	14	439	24	18	14
d-dimer, ng/mL	0–500	161	89	101	>3500	150	97

*ALT, alanine aminotransferase; AST, aspartate aminotransferase; SARS-CoV-2, severe acute respiratory syndrome coronavirus 2. †Contact 2 had 4 serial negative throat swabs for SARS-CoV-2, and negative influenza A, influenza B, respiratory syncytial virus, parainfluenza virus, adenoviridae, Epistein-Barr virus, cucumber mosaic virus, mycoplasma, and chlamydia results. He had elevated AST and ALT and was negative for hepatitis A, B, C, and E; he had no jaundice or gastrointestinal symptoms. His AST and ALT returned to normal after 9 days of treatment with glycyrrhizinate 50 mg 3 times daily and vitamin C (0.2 g 3×/d). ‡Contact 3 had an elevated D-dimer level without anemia, bleeding, or evidence of a coagulopathy. She received vitamin C (0.2 g 3 ×/d). After the SARS-CoV-2 nucleic acid (throat swab) test was negative, her D-dimer level returned to normal (111 ng/mL).

All 5 household contacts of patient 1 had laboratory evidence of SARS-CoV-2 infection but remained asymptomatic throughout the period of observation (February 11–March 1) (Figure, panel A). All household contacts who had throat swab specimens tested for SARS-CoV-2 were positive by PCR except for contact 2, who tested negative on 4 consecutive throat swab specimen tests for SARS-CoV-2 but whose stool specimen was positive for SARS-CoV-2; contact 2 also had elevated liver enzymes but no jaundice. Contact 3 had an elevated D-dimer level. These abnormal laboratory values resolved during observation (Table) and were not associated with clinical illness in either patient. Patient 1 and contacts 2 and 4 also had abnormal chest computed tomography scans consistent with SARS-CoV-2 infection (Figure, panel B).

Contact 1 underwent 11 serial throat swabs for SARS-CoV-2. Her case demonstrates the challenges of clinical interpretation qRT-PCR results for SARS-CoV-2. On 2 separate occasions, she had 2 consecutive negative results on throat swab specimens for SARS-CoV-2, only to revert back to having a throat swab specimen positive for SARS-CoV-2 (Figure, panel A). Contact 1 was the only family member who underwent serologic tests, which demonstrated low B lymphocyte counts but no detectable SARS-CoV-2–specific IgM or IgG. We cannot determine the cause or clinical significance of the lack of a detectable antibody response in contact 1 in our study, which differs from findings reported in other studies (*6*). The immunologic response after asymptomatic SARS-CoV-2 infection requires further study.

**Figure:**
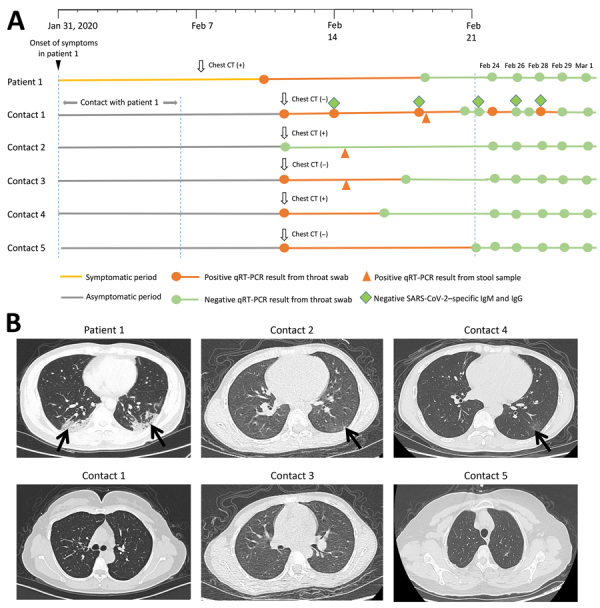
Timeline and CT images associated with a cluster of SARS-CoV-2 infections in a single household, Wuhan China. A) Timeline of key events, including laboratory tests, associated with SARS-CoV-2 infections in the index patient and 5 asymptomatic household contacts. B) Abnormal chest CT scans showing features consistent with SARS-CoV-2 infection (arrows) observed in the index patient and 2 household contacts (top row), compared with normal CT scans among the 3 other household contacts (bottom row). CT, computed tomography; qRT-PCR, quantitative real-time reverse transcription PCR; SARS-CoV-2, severe acute respiratory syndrome coronavirus 2.

A likely source of infection for the 5 asymptomatic contacts was patient 1. Contact 1 had no patient contact and no known contact with COVID-19–positive co-workers, and contacts 2, 3, 4, and 5 were at their home in Wuhan and had no other substantial human contact during the period when they likely were infected. We identified no other likely source of infection. Our study could not determine the method of transmission between family contacts, but we did note the potential for respiratory transmission (e.g., through droplets), fecal–oral transmission, or both.

An early report from China on 72,314 COVID-19 cases found that only 1% of SARS-CoV-2 infections were asymptomatic; however, asymptomatic close contacts were not routinely tested in that study (*7*). In our study, all 5 household contacts of a physician diagnosed with COVID-19 had laboratory evidence of infection but remained asymptomatic. This finding is consistent with emerging evidence that suggests that a substantial proportion of SARS-CoV-2 infections are asymptomatic (*1*,*8*,*9*).

In summary, this single-household study found a high attack rate for asymptomatic SARS-CoV-2 infection among the immediate family members of a symptomatic COVID-19 case-patient. The extent to which asymptomatic SARS-CoV-2 infections contribute to overall disease transmission is still unknown and warrants further study. We believe the potential for fecal–oral transmission also warrants investigation (*10*). Moreover, our experience indicates that screening symptomatic contacts with a single throat swab test for SARS-CoV-2 might lead to an underestimate of the rate of infection and that asymptomatic persons can repeatedly revert between positive and negative PCR results on throat specimens.

## References

[1] Sutton D, Fuchs K, D’Alton M, Goffman D. Universal screening for SARS-CoV-2 in women admitted for delivery. N Engl J Med. 2020;NEJMc2009316; Epub ahead of print. 10.1056/NEJMc2009316 32283004 PMC7175422

[2] Chan JF, Yuan S, Kok KH, To KK, Chu H, Yang J, et al. A familial cluster of pneumonia associated with the 2019 novel coronavirus indicating person-to-person transmission: a study of a family cluster. Lancet. 2020;395:514–23. 10.1016/S0140-6736(20)30154-9 31986261 PMC7159286

[3] Bai Y, Yao L, Wei T, Tian F, Jin DY, Chen L, et al. Presumed asymptomatic carrier transmission of COVID-19. JAMA. 2020;323:1406. 10.1001/jama.2020.2565 32083643 PMC7042844

[4] Ye F, Xu S, Rong Z, Xu R, Liu X, Deng P, et al. Delivery of infection from asymptomatic carriers of COVID-19 in a familial cluster. Int J Infect Dis. 2020;S1201-9712(20)30174-0; [Epub ahead of print]. 32247826 10.1016/j.ijid.2020.03.042 PMC7129961

[5] Hu Z, Song C, Xu C, Jin G, Chen Y, Xu X, et al. Clinical characteristics of 24 asymptomatic infections with COVID-19 screened among close contacts in Nanjing, China. Sci China Life Sci. 2020 Mar 4 [Epub ahead of print]. 10.1007/s11427-020-1661-4 PMC7088568 32146694

[6] Du Z, Zhu F, Guo F, Yang B, Wang T. Detection of antibodies against SARS-CoV-2 in patients with COVID-19. J Med Virol. 2020; Epub ahead of print. 10.1002/jmv.25820 32243608 PMC7228383

[7] Wu Z, McGoogan JM. Characteristics of and important lessons from the coronavirus disease 2019 (COVID-19) outbreak in China: summary of a report of 72,314 cases from the Chinese Center for Disease Control and Prevention. JAMA. 2020;323:1239. 10.1001/jama.2020.2648 32091533

[8] Mizumoto K, Kagaya K, Zarebski A, Chowell G. Estimating the asymptomatic proportion of coronavirus disease 2019 (COVID-19) cases on board the Diamond Princess cruise ship, Yokohama, Japan, 2020. Euro Surveill. 2020;25. 10.2807/1560-7917.ES.2020.25.10.2000180 32183930 PMC7078829

[9] Nishiura H, Kobayashi T, Suzuki A, Jung SM, Hayashi K, Kinoshita R, et al. Estimation of the asymptomatic ratio of novel coronavirus infections (COVID-19). Int J Infect Dis. 2020;S1201-9712(20)30139-9; Epub ahead of print. 10.1016/j.ijid.2020.03.020 32179137 PMC7270890

[10] Chen Y, Chen L, Deng Q, Zhang G, Wu K, Ni L, et al. The presence of SARS-CoV-2 RNA in feces of COVID-19 patients. J Med Virol. 2020; Epub ahead of print. 10.1002/jmv.25825 32243607

